# Translin and Trax differentially regulate telomere-associated transcript homeostasis

**DOI:** 10.18632/oncotarget.9278

**Published:** 2016-05-10

**Authors:** Natalia Gomez-Escobar, Nasser Almobadel, Othman Alzahrani, Julia Feichtinger, Vicente Planells-Palop, Zafer Alshehri, Gerhard G. Thallinger, Jane A. Wakeman, Ramsay J. McFarlane

**Affiliations:** ^1^ North West Cancer Research Institute, School of Medical Sciences, Bangor University, Bangor, Gwynedd, United Kingdom; ^2^ Computational Biotechnology and Bioinformatics Group, Institute of Molecular Biotechnology, Graz University of Technology, Graz, Austria; ^3^ Omics Center Graz, BioTechMed Graz, Graz, Austria

**Keywords:** telomeres, Translin, Trax, C3PO, TERRA, Chromosome Section

## Abstract

Translin and Trax proteins are highly conserved nucleic acid binding proteins that have been implicated in RNA regulation in a range of biological processes including tRNA processing, RNA interference, microRNA degradation during oncogenesis, spermatogenesis and neuronal regulation. Here, we explore the function of this paralogue pair of proteins in the fission yeast. Using transcript analysis we demonstrate a reciprocal mechanism for control of telomere-associated transcripts. Mutation of *tfx1*^+^ (Trax) elevates transcript levels from silenced sub-telomeric regions of the genome, but not other silenced regions, such as the peri-centromeric heterochromatin. In the case of some sub-telomeric transcripts, but not all, this elevation is dependent on the Trax paralogue, Tsn1 (Translin). In a reciprocal fashion, Tsn1 (Translin) serves to repress levels of transcripts (TERRAs) from the telomeric repeats, whereas Tfx1 serves to maintain these elevated levels. This reveals a novel mechanism for the regulation of telomeric transcripts. We extend this to demonstrate that human Translin and Trax also control telomere-associated transcript levels in human cells in a telomere-specific fashion.

## INTRODUCTION

The faithful segregation of chromosomes during the eukaryotic cell cycle is a multifaceted event, failures in which can drive diverse human diseases, including cancers [1]. Protection of the termini of linear eukaryotic chromosomes, the telomeres, during this process is essential to prevent nucleolytic chromosomal degradation [2]. Telomere maintenance is normally mediated by a specific DNA polymerase termed telomerase, which is capable of extending telomeric DNA independently of the major genomic replisome [3, 4]. Telomere length control provides a molecular limiter on cellular proliferative potential as telomerase activity is repressed in most healthy somatic cells. Without length homeostasis being maintained, telomeres will shorten with each division, ultimately resulting in cellular senescence [3, 5]. Proliferating cancer cells must override this senescence pathway, either via the activation of telomerase, chromosomal circularization or activation of alternative telomere maintenance pathways [6]. Telomeres also contribute to the correct chromosomal architecture within the nucleus [7] and telomere dysfunction has been associated with a range of human genetic diseases [8].

Telomeric DNA is highly repetitive in nature and associates with a plethora of telomere regulatory proteins to form a unique nucleoprotein structure [2, 3, 9]. Chromosomal regions adjacent to the telomeres are often subjected to heterochromatic transcriptional silencing [4, 10, 11] although long noncoding telomeric repeat-containing RNA (TERRA) is transcribed from telomeric DNA repeats [12-16]. The function(s) and regulators of TERRAs remains poorly defined, but TERRAs have been implicated in a range of telomere-associated processes, including DNA damage response [12-16].

The human protein Translin was first identified by virtue of its ability to bind to the breakpoint junctions of chromosomal translocations in lymphoid malignancies [17] and other genetic diseases [18]. It was independently identified in the mouse as the testis-brain RNA binding protein [19]. Translin is highly conserved in eukaryotes and archaea and has a paralogue binding partner Trax (Translin-associated protein X) [18]. The Translin-Trax complex has the ability to bind to nucleic acid, with a bias towards RNA, and it possesses RNA nucleolytic activity [18, 20-23]. DNA sequence binding preferences has led to the suggestion that Translin may function in telomere regulation, although to date there is no direct evidence for this [24]. Telomeres directly interact with proteins associated with homologous recombination and DNA double-strand break (DSB) repair [25], and Trax has recently been demonstrated to have a role in the murine DNA damage response via association with the ATM pathway [26]. Whilst a role for Translin in this response has not yet been found, Translin function has also been linked to the mammalian DNA damage response [27].

Since their initial identification, Translin and Trax have been implicated in a wide range of nucleic acid processing pathways, including tRNA maturation and mRNA regulation both in spermatogenesis and neuronal dynamics, with mutations in the Translin binding site of *BDNF* (brain-derived neurotrophic factor) mRNA resulting in memory deficits and psychiatric disorders [18,28,29]. Recently, *Drosophila* and mammalian Translin and Trax were revealed to be the constituents of the C3PO complex [22, 30-32], which is responsible for assisting the removal of the passenger RNA strand from small interfering RNAs involved in Argonaute-dependent RNA-induced transcriptional silencing [33]. In most activities studied to date, Translin and Trax have been shown to function in unison, and the maintenance of Trax stability is a conserved function of Translin [18, 27]. Importantly, a direct role in oncogenesis has recently been reported for Translin and Trax in cancers that are haploinsufficient for Dicer, as they degrade pre-microRNAs that would be processed to microRNAs by a full Dicer complement to maintain tumour suppression [34]. This has led to the suggestion that Translin and Trax could provide drug targets in Dicer haploinsufficient tumours [34].

Here we reveal previously unknown roles for Translin and Trax in controlling homeostasis of distinct telomere-associated transcripts in both fission yeast and human cells. Moreover, we demonstrate that Translin and Trax can act independently, indicating that they do not function solely as a heteromeric complex, but have interrelated roles in controlling telomere-associated transcript.

## RESULTS

### Trax, but not Translin, represses sub-telomeric transcript levels

**Figure 1:**
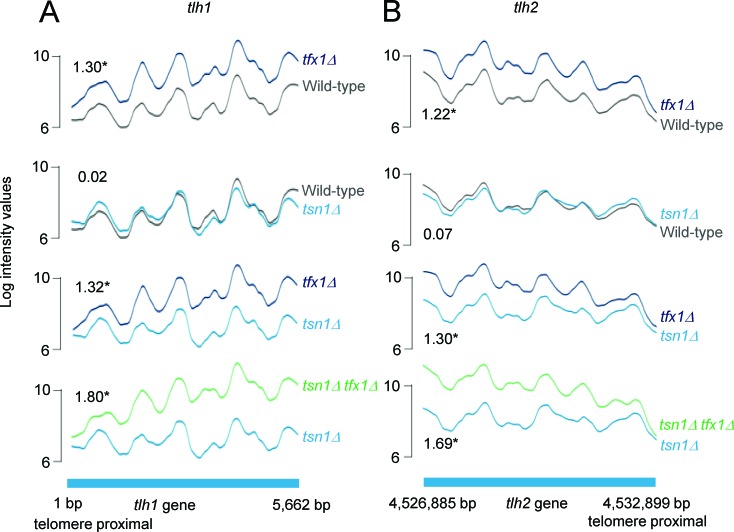
Tfx1 (Trax), but not Tsn1 (Translin) regulates sub-telomeric transcript levels in *S. pombe* Both images show relative transcript levels obtained from tiling microarrays. **A.** *tlh1* gene transcripts are elevated in a *tfx1*Δ mutant, but not in *tsn1*Δ mutant. Elevation is maintained in a *tfx1Δ tsn1*Δ double mutant. Base pair (bp) designation represents the nucleotide annotation position for *S. pombe* chromosome 1 (*tlh1* ORF is on the reverse strand, hence reverse strand profile is shown). **B.** A similar profile is seen for the other annotated sub-telomeric *tlh* gene, *tlh2*. Fold change values are given within the plots (* = P<0.05).

The extensive conservation of Translin and Trax indicate they serve a fundamentally important biological role. Despite this, deletion of both the Translin (*tsn1*^+^) and Trax (*tfx1*^+^) genes of the fission yeast *Schizosaccharomyces pombe* does not result in any readily detectable phenotypic change [35, 36]. Given the finding that Translin and Trax regulate RNA dynamics in other organisms, we set out to determine whether *S. pombe* Tsn1 and Tfx1 are involved in transcript regulation. To do this, we used tiling arrays to analyse the transcriptome and to identify any changes when *tsn1^+^* and *tfx1^+^* are mutated. Comparison of the wild-type and *tsn1*^+^ transcripts reveals no statistically meaningful difference. However, comparison of the wild-type and *tfx1*Δ strains revealed one significant change: an up-regulation of sub-telomeric *tlh* gene transcripts in the *tfx1*Δ mutant (Figure 1). It should be noted that the annotated DNA sequences for *tlh1* and *tlh2* genes are 100% identical so we cannot rule out a *tlh1*- or *tlh2*-only dysregulation (i.e. a specific sub-telomeric region). The double *tsn1*Δ *tfx1*Δ mutant exhibits the same *tlh* transcript elevation seen in the *tfx1*Δ single mutant (Figure 1). Not only is the elevation of the *tlh* transcripts the first measurable phenotype of a *tfx1*Δ mutant, it also reveals a functional separation between Tfx1 and Tsn1 in *S. pombe. S. pombe* Tsn1 functions to stabilize Tfx1 [35] and in the absence of Tsn1 there are considerably reduced levels of Tfx1 [35]. The *tlh* transcript data indicate that the low levels of Tfx1 in the *tsn1*Δ background remain sufficient to maintain *tlh* transcript repression.

**Figure 2:**
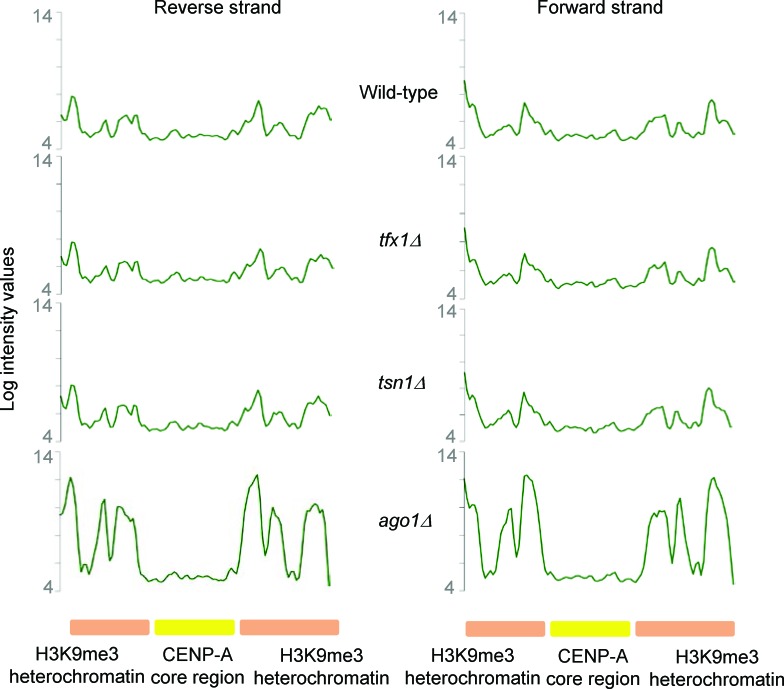
Centromeric transcript levels remain unaltered upon loss of Tsn1 or Tfx1 Centromeric transcript levels remains unaltered in either *tfx1*Δ or *tsn1*Δ mutants (middle two profiles) compared to the wild-type (top profile). The profile for an *ago1*Δ mutant is given as a control for a desilencing mutant (bottom profile). The approximate spread of heterochromatic and centromere core regions are given (bottom line). The profiles are for the forward (right) and reverse (left) strand of *cen1*. The *S. pombe* nucleotide coordinates shown for *cen1* are chromosome 1: 3,754,000 – 3,790,000. Both strands for *cen2* and *cen3* show similar inactivation in *tfx1*Δ and *tsn1*Δ mutants and are given in Figure S1 and S2 respectively.

There are four *tlh* paralogues within the *S. pombe* genome (although only two are currently annotated and have their transcription measured using the tiling arrays; C. Norbury, personal communication). One paralogue is located within the sub-telomeric regions of each of the four telomeres for *S. pombe* chromosomes 1 and 2 (*S. pombe* has three chromosomes and the sub-telomeric regions of chromosome 3 are unique as they consist of rDNA repeats). The *tlh* genes are *BLM* gene orthologues of unknown function, although they have been implicated in recovery from telomerase loss crisis and they are normally subjected to transcriptional silencing [37]. Given the fact that sub-telomeric regions are governed by RNA interference-mediated transcriptional silencing, which also regulates the heterochromatic transcriptional silencing in the outer repeat regions of the three *S. pombe* centromeres [10], we carefully examined transcription from these centromeric repeat regions in the *tsn1*Δ and *tfx1*Δ mutants. No measurable change in transcript levels between mutants and wild-type was measured for either DNA strand (Figure 2, Figure S1-S2). Collectively, these data indicate that Tfx1, but not Tsn1 functions in sub-telomeric, but not centromeric, transcript regulation.

### Loss of Tfx1 suppresses the requirement for Ago1

**Figure 3:**
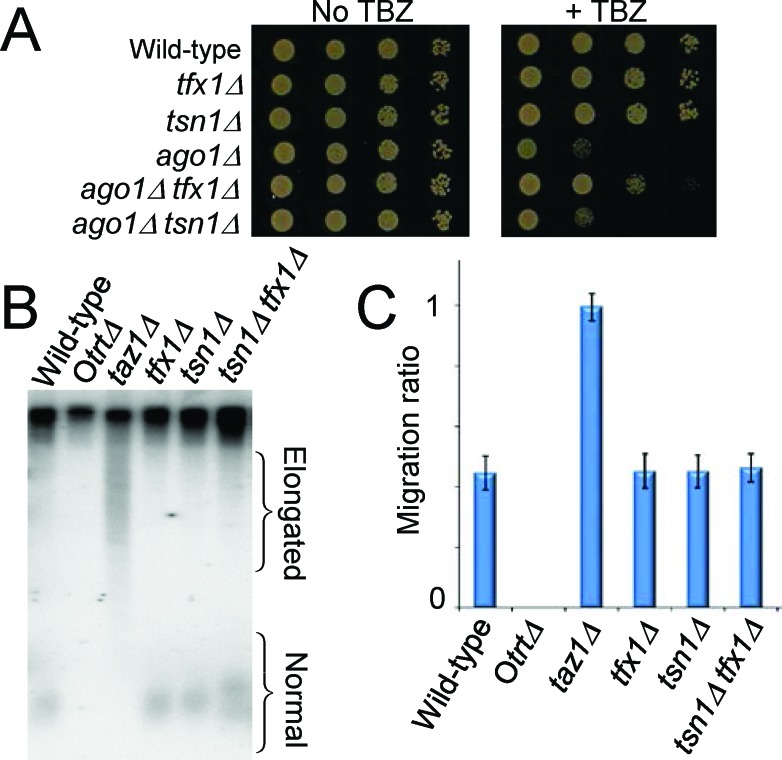
Loss of Tfx1 results in a telomere-defective phenotype, but telomere length is unaltered **A.** Suppression of TBZ sensitivity of an *ago1*Δ mutant is a feature of telomere regulator proteins [38]. The *tfx1*Δ mutant, like the *taz1*Δ mutant [38] (Figure S3), partially suppresses the *ago1*Δ TBZ sensitivity, whereas a *tsn1*Δ mutation does not. **B.** Southern blot probed with a telomere-specific probe showing that both *tfx1*Δ and *tsn1*Δ mutants have telomeres with lengths similar to the wild-type. The *taz1*Δ mutant had highly elongated telomeres. The *Otrt*Δ strain has circular chromosomes without telomeres and serves as a control for the probe specificity. **C.** Quantification of telomere fragment migration ratios demonstrates no statistically significant difference between the mean telomere lengths of the *tfx1*Δ, *tsn1*Δ and wild-type strains. Mean migration values for at least four repeats were obtained. Pairwise Student's *t-*tests were conducted between all telomere containing strains (i.e. not the *Otrt* strain) and the wild-type. All *P* values were > 0.05 with the exception of the wild-type *vs. taz1*^+^ analyses which was < 0.01. Error bars are standard deviations.

Similar to the loss of Tfx1, the loss of the telomere regulator Taz1 [38] has also been demonstrated to elevate the *tlh* transcript levels [39]. Moreover, loss of Taz1 function has been shown to partially suppress sensitivity to the microtubule destabilizing drug thiabendazole (TBZ) of cells mutated in the RNA interference regulator gene *ago1*^+^ [40] (Figure S3). It has been postulated that this suppression occurs due to heterochromatin factors mediating the sub-telomeric silencing being ‘released’ from the sub-telomeric regions to relocate and enhance the centromeric function that is compromised due to loss of Ago1 [40]. To determine whether loss of Tfx1 results in a similar phenotypic outcome as loss of the known telomere regulator Taz1, we generated *ago1*Δ *tfx1*Δ and *ago1*Δ *tsn1*Δ double mutants and tested their response to TBZ. Consistent with the *tlh* transcript up-regulation data (Figure 1), Figure 3A shows that loss of Tfx1, but not Tsn1 partially suppresses the need for Ago1, which is the same for the suppression reported upon loss of Taz1 [40] (Figure S3), consistent with a functional link for Tfx1 to telomeric regulation.

### Tfx1 and Tsn1 do not regulate telomere length

Taz1 is required to limit telomere length and in the absence of Taz1 telomeres become highly elongated [38]. Given the phenotypic similarities between *taz1*Δ and *tfx1*Δ mutants, we wished to determine whether Tfx1 also contributed to telomere length regulation. To address this, Southern blot analysis was carried out to compare telomere lengths between a *taz1*Δ, *tsn1*Δ and *tfx1*Δ mutants (and the *tfx1*Δ *tsn1*Δ double mutant). *tsn1*Δ and *tfx1*Δ single mutants had mean telomere lengths indistinguishable from the wild-type, whereas telomeres in the *taz1*Δ mutant were greatly elongated (Figure 3B/C). These data indicate that the de-silencing of the *tlh* genes seen in the *tfx*1Δ mutant is not related to telomere length dysregulation, as it is in the *taz1*Δ mutant.

### Tsn1 and Tfx1 differentially regulate telomeric transcripts

**Figure 4:**
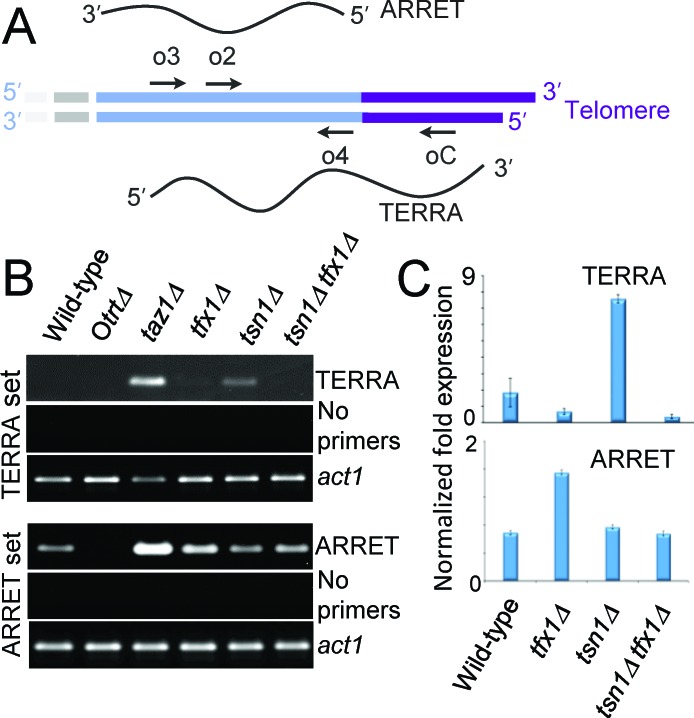
Translin and Trax regulate telomere-associated transcripts in fission yeast cells **A.** A schematic of an *S. pombe* telomere showing the telomere (purple) and the sub-telomeric region (light blue; previously referred to as STE) [42]. The ARRET and TERRA transcripts are aligned to their approximate template location. Arrows indicate primer positions used for first strand cDNA synthesis and RT-PCR (for TERRAs cDNA priming used oC, PCR was primed using o2/o4; for ARRETs cDNA priming used o3 and PCR was primed using o2/o4; primer sequences and designations are derived from Greenwood and Cooper [42]). **B.** Agarose gels showing analytical RT-PCR for TERRAs (first strand oC) and ARRETs (first strand o3). No primer controls indicate no first strand cDNA primers, demonstrating that PCR products are not due to endogenous first strand priming. RT-PCT for the *act1*^+^ gene transcripts are given as a positive control. **C.** RT-qPCR for TERRAs and ARRETs in *tsn1*Δ and *tfx1*Δ cells. Error bars represent standard deviation. Student's *t*-test pairwise comparison were carried out for mean values between the wild-type *vs*. mutants. In all cases *P* values were > 0.05 with the exceptions of wild-type *vs. tsn1*^+^ for TERRA levels (upper set) and wild-type *vs. tfx1*^+^ for ARRET levels (lower set) where *P* values were < 0.01.

TERRA production from telomeric DNA template is a conserved feature of telomeres in both humans and fission yeast [12-16]. Other sub-telomeric transcripts, termed ARRETs have also been detected in *S. pombe* for the sub-telomeric regions on chromosomes 1, 2 and occasionally 3 [41, 42]. ARRET and TERRA sequences are not covered on the tiling arrays used in this study. To further confirm the regulation of sub-telomeric transcripts by Tfx1, we examined ARRET (immediately sub-telomeric regions) levels in *tfx1*Δ and *tsn1*Δ strains using RT-PCR/RT-qPCR [43]. Loss of Taz1 gives elevated ARRET levels, consistent with activation of *tlh* genes in the absence of Taz1 [39, 42]. Loss of Tfx1 resulted in an approximately two-fold elevation in ARRETs, again, consistent with the *tlh* transcripts (Figure 4). However, unlike the *tlh* transcript elevation, additional mutation of *tsn1*^+^ results in a reduction of ARRET transcripts back to wild-type levels, indicating an interplay between Tfx1 and Tsn1 for ARRET regulation, but not *tlh* transcript regulation (Figure 1, Figure 6); so, in the absence of Tfx1, Tsn1 is required to maintain elevated ARRET levels.

Taz1-defficient cells also exhibit elevated levels of TERRAs [42]. Telomeric DNA is not extended in the *tfx1*Δ mutant (Figure 3B/C) and so we wished to address whether the regulation of telomere-associated transcripts extended into the TERRA encoding telomeric regions. To do this, we analysed TERRA levels in the various mutants. Using RT-PCR/RT-qPCR we could only detect very limited TERRA levels in the wild-type (Figure 4). However, both *taz1*Δ and *tsn1*Δ mutants exhibited clearly elevated levels of TERRA (Figure 4), whereas levels in the *tfx1*Δ mutant were indistinguishable from the wild-type. This surprise finding indicates that Tsn1 is required for suppression of TERRA levels whereas Tfx1 is required for suppression of sub-telomeric transcripts (*tlh* genes and ARRETs). Interestingly, additional mutation of *tfx1*^+^ in the *tsn1*Δ background results in restoration of TERRA levels to those of the wild-type indicating that in the absence of Tsn1, Tfx1 stabilizes the elevated levels of TERRA (Figure 4). So, importantly, these findings indicate an inverted reciprocal relationship between Tfx1 and Tsn1 for sub-telomeric and telomeric transcript regulation, respectively. Upon loss of Tfx1, ARRETs, but not TERRAs, are elevated in a Tsn1-dependent fashion, and upon loss of Tsn1, TERRAs, but not ARRETs, are elevated in a Tfx1-dependent fashion. The requirement for Tsn1 to maintain elevated sub-telomeric transcripts only applied to the ARRETs and not the *tlh* genes indicating a positional constraint on this relationship, possibly governed by proximity to the telomeric DNA where Tsn1 functions to suppress TERRA transcript levels. *S. pombe* also produces other telomeric and sub-telomeric transcripts (termed ARIAs and α-ARRETs respectively) [41, 42], but we could not distinguish RT-PCR products of these transcripts from transcripts produced by first strand cDNA primed by endogenous priming; for both TERRA and ARRET analysis lack of first strand primers produced no PCR products and so problematic endogenous priming was eliminated (Figure 4).

### Translin and Trax regulate human TERRA levels

**Figure 5:**
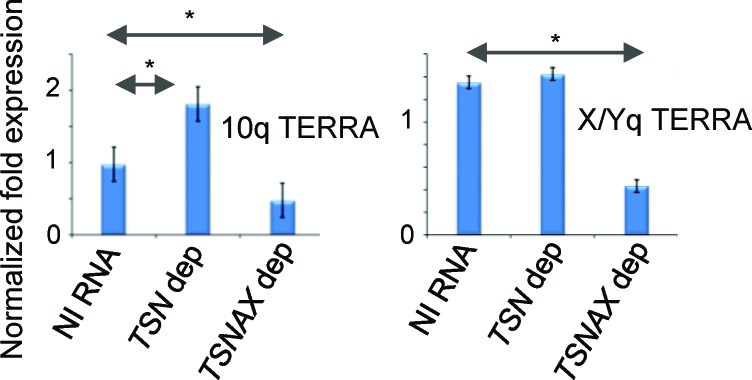
Human Translin and Trax influence TERRA levels differentially for distinct telomeres RT-qPCR for human TERRAs for the telomeres of the q arm of chromosome 10 (left) and the q arm of the sex chromosome (X/Y; right) in TSN and TSNAX depleted SW480 cells. NI RNA = non-interfering RNA. Error bars represent standard deviations. Student's *t*-test pairwise comparison were carried out for mean values between NI RNA treated cells and siRNA depleted cells. Only comparisons marked with * showed statistical significance (*P* < 0.05).

TERRA was originally detected in mammals [44, 45]. To explore whether the role of Translin in suppressing TERRA levels was conserved, we measured TERRAs for the q arm of chromosome 10 and the sex chromosomes (X/Y) [46] in human SW480 cells following siRNA depletion of either *TSN1* (Translin) or *TSNAX* (Trax) expression (Figure S4). Figure 5 shows that TERRA levels were elevated in TSN-depleted cells, but not in TSNAX-depleted cells for the 10q telomere. The TSNAX-depleted cells showed reduced TERRA levels for all telomeres tested.

These findings indicate that the separation of roles for Translin and Trax in TERRA regulation is conserved from *S. pombe* to some telomeres in humans. The 10q TERRA data indicate that Translin serves to suppress levels of some human TERRAs, whereas Trax serves to stabilize/maintain their levels, raising the possibility of a reciprocal TERRA stabilization mechanism at play. The same relationship was not readily observed for the q arm of the sex chromosomes, where depletion of TSN did not result in elevated X/Yq TERRAs, but depletion of TSNAX reduced TERRAs (Figure 5). These data indicate a more complex picture in human cells, with the potential for a telomere-specific requirement for distinct TSN and TSNAX functions.

## DISCUSSION

### Translin and Trax function to regulate telomere transcripts

Translin and Trax influence distinct biological processes in a species- and tissue-specific manner [18]. The majority of the biological roles appear to require the modulation of RNA (not DNA) molecules, including mRNAs, tRNA precursors, microRNAs and siRNA passenger strands [18], although the functional significance of chromosomal translocation breakpoint junction binding capability remains unclear [17]. Here we demonstrate that Translin and Trax control telomere-associated transcript abundance, but do so in distinct fashions. That these factors function in such a fundamental process as telomere transcript regulation, yet their disruption does not result in significant perturbation of proliferative potential, in fission yeast at least, indicates that there is a degree of tolerance for alteration of telomeric transcript levels. In mammalian cells loss of Translin and Trax function has been linked to reduced cell proliferation [47-49], but this may relate to disruption of non-telomeric functions in these cells. Given that dysregulation of telomeric transcripts is the only defect in *tsn1*Δ and *tfx1*Δ mutants of *S. pombe*, this makes it an excellent system in which to study this important function of these proteins in the absence of perturbation of other biological roles.

TERRAs control a range of telomere-associated functions, including regulation of telomerase activity, telomere length control, telomeric heterochromatinization and cellular differentiation [12-16]. TERRA has both positive and negative influences upon telomere length regulation, with cell cycle and tissue-specific influences [16]. In budding yeast, loss of Rat1 ribonuclease processing results in accumulation of TERRAs and inhibition of telomerase leading to telomere shortening [50]. Whilst we show here that loss of *tsn1* also results in TERRA accumulation, this alone does not appear to be sufficient for measurable telomere length alteration or reduction in cell viability. This function of *tsn1* seems to be conserved, as depletion of human TSN results in elevation of TERRA from the q arm telomere of chromosome 10, although this same effect was not seen for the q arm of X/Y, which might reflect a requirement for distinct levels of TSN (siRNA does not cause full cellular depletion) or a telomere specificity for TSN function. Interestingly, a recent systematic approach to identify telomere DNA and TERRA interacting proteins did not identify TSN or TSNAX. This suggests that telomere-associated transcriptome regulation by TSN and/or TSNAX in humans is likely mediated by an indirect and/or transient/labile association [51]. In *S. pombe*, Translin (Tsn1) only forms weak interactions with other non-Trax (Tfx1) proteins [52], and possibly other macromolecules, and no interactions have been demonstrated with known telomere regulators.

### Distinct and joint functions for Translin and Trax

**Figure 6:**
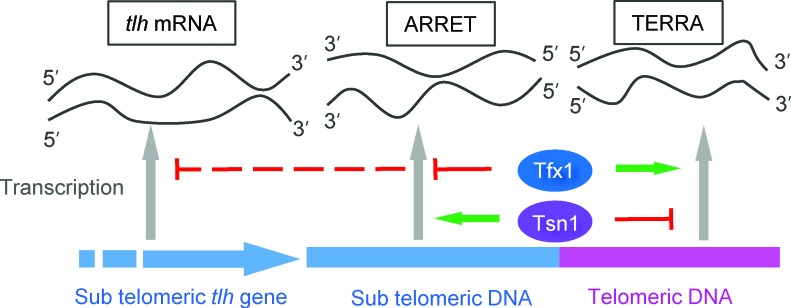
Model for the mechanism of telomere-associated transcript differential control by Tfx1 and Tsn1 in *S. pombe* Tfx1 serves to repress sub-telomeric ARRETs and *tlh* transcripts (upper red full/broken lines), but stabilizes (upper green arrow) elevated levels of TERRAs seen in the *tsn1*Δ mutant. Tsn1 plays a reciprocal role, normally suppressing TERRA levels (lower red line), but stabilizing (lower green arrow) the elevated ARRET levels (but not the elevated *tlh* transcript levels) seen in the *tfx1*Δ mutant.

Almost all studies to date, revealing a biological role for Translin and Trax, have demonstrated a close functional association between the two [18]. Here, we show both a joint and separate functional association between these paralogues. Firstly, the transcript up-regulation for sub-telomeric regions only occurs when *tfx1^+^* is mutated but not upon *tsn1*^+^ mutation. The elevation of ARRET levels is *tsn1*-dependent (Figure 4), but the *tlh* transcripts remain elevated in the *tsn1Δ tfx1*^+^ double mutant (Figure 1), indicating there may be a transcript/positional specificity to this regulation. Conversely, the elevation of TERRA levels in the *tsn1*^+^ mutant is *tfx1*-dependent. These findings indicate that there is a reciprocal control mechanism operating between Tsn1 and Tfx1 to maintain telomere-associated transcriptional homeostasis (Figure 6). Previously, it has been demonstrated that mouse Trax can inhibit the binding of mouse Translin to mRNA [53], and a similar direct regulation might be occurring here to drive the inter paralogue modulation mechanism. Additionally, when *tsn1^+^* is mutated, Tfx1 levels become depleted [35], so the residual Tfx1 found in the *tsn1*^+^ mutant must be sufficient to maintain a functional role for telomere transcript level regulation.

Trax function has recently been linked to the DNA damage response [26] and TERRAs have been linked to the telomeric DNA damage response [12-16]. We previously found no altered sensitivity of the *S. pombe tfx1*^+^ (or *tsn1*Δ) mutant to a wide range of DNA damaging agents [34], so there is no current evidence to link this phenomenon to telomeric DNA damage tolerance in *S. pombe*.

The finding that Translin and Trax control telomeric transcript homeostasis adds a new dimension to our understanding of these conserved nucleic acid regulators. It has recently been proposed that Translin and Trax may provide anti-cancer therapeutic targets [34]; however, before small molecule inhibitors are developed and applied to the clinical setting it is of fundamental importance that we fully elucidate the normal function of this protein pair to ensure that inhibition of their function does not adversely influence non-diseased tissues.

## MATERIALS AND METHODS

### *S. pombe* strains and media

A list of strains used in this study and their genotypes are shown in Table S1. Media, standard growth conditions, *S. pombe* transformations and genetic analyses were as described by Forsburg and Rhind [54]. All gene deletions in strains were created as described by Bähler and co-workers [55].

### *S. pombe* DNA extraction and Southern blotting

DNA extraction was performed as described in Forsburg and Rhind [54]. After digestion with *Apa*I (New England Biolabs), 15 μg of DNA was separated on a 1.2% agarose gel, transferred to positively charged nylon membrane (GeneScreen *Plus*) and hybridized to a radioactively labelled probe at 42°C for 18 hours in Church Gilbert buffer. Oligonucleotide probe corresponding to the telomeric G-strand was 5′-end labelled with T4 polynucleotide kinase (New England Biolabs) in the presence of [γ-^32^P]ATP. The probe sequence was 5′- GGGTTACAGGTTACAGGTTACA-3′. After hybridization, membranes were washed three times in 6 x SSC/0.1% SDS at room temperature. Radioactive signals were detected using a Molecular Imager FX (Bio-Rad) and analysed using Quantity One (Bio-Rad). Migration ratio was calculated as the ratio of the distance migrated for the 2 kb marker as a fraction of the distance migrated by the telomere probe signal midpoint as determined by Quantity One. Mean values were obtained from four independently generated blots.

### Human cell cultures

Human SW480 cell line was obtained from the European Collection of Cell Culture. They were grown in Invitrogen Dulbeco's Modified Eagle's medium (DMEM+Glutamax) supplemented with 10% foetal bovine serum (Life Technologies) in a 37°C incubator with 5% CO_2._ SW480 cells were authenticated prior to use using LGC Standards Cell Line Authentication service (tracking number 710418378). Cells were checked for mycoplasma at regular intervals using the LookOut® Mycoplasma Detection Kit (Sigma Aldrich; MP0035).

### RNA extraction and reverse transcription PCR

Total RNA was isolated from *S. pombe* cells using the MasterPure™ Yeast RNA Purification Kit (Epicentre) following the manufacturer's instructions and treated with RNase-free DNase (Promega). Total RNA was extracted from human SW480 cells using TRIzol (Life Technologies) as described by Feichtinger and co-workers [56]. 1 μg of RNA was used as template in first strand synthesis by Superscript III (Invitrogen). The RNA was denatured in the presence or absence of primer at 90°C for 1 minute followed by cooling to 55°C before adding the reverse transcriptase mix and incubating for 50 minutes. A volume of 2 μl of cDNA was PCR amplified using MyTaq™ Red Mix (Bioline). PCR with subtelomeric primers was carried out for 3 minutes at 95°C, then 35 cycles of 95°C for 30 seconds, 62°C for 20 seconds, 72°C for 20 seconds, followed by 72°C for 5 minutes. The same cycling conditions were used for *act1*, except that an annealing temperature of 58°C was used for 25 cycles.

RNA reversed transcribed with Superscript III was also used for TERRA qRT-PCR experiments. cDNA was amplified using the GoTaq® qPCR Master Mix (Promega) on a CFX96 real-time system (Bio-Rad) following the manufacturer's protocol. Sequences of all primers used in the study are given in Table S2.

### Whole transcriptome microarray analysis

Appropriate strains were cultured to an absorbance of 0.5 at 600 nm and RNA was collected from cells using the hot phenol method. The RNA was processed as described by Kowalik and co-workers [57] following the processed described in the GeneChip Whole Transcript Double-Stranded Target Assay Manual from Affymetrix using the GeneChip *S. pombe* Tiling 1.0FR. Data processing is described in the Supplemental methods.

### TSN and TSNAX depletion in SW480 cells

SW480 cells were transfected with pre-designed siRNAs directed to *TSNAX* (Qiagen, SI00751982) and *TSN* (Qiagen, SI03650318) using HiPerFect Transfection Reagent (Qiagen). After three hits, each lasting 24 hours, RNA was extracted using RNeasy Mini Kit (Qiagen) and reverse transcribed using the SuperScript III First-Strand Synthesis System (Invitrogen) in the presence of oligo dT cDNA was amplified using the GoTaq® qPCR Master Mix (Promega) on a CFX96 real-time system (Bio-Rad) following the manufacturer's protocol. Primer sequences are provided in Table S2.

## SUPPLEMENTARY MATERIAL FIGURES AND TABLES


