# Supplementary material for: Translin and Trax differentially regulate telomere-associated transcript homeostasis
Source: Oncotarget. 2016 May 10;7(23):33809–20. doi: 10.18632/oncotarget.9278 (PMC5085120; doi:10.18632/oncotarget.9278)
Supplement: Supplementary file 1 [file oncotarget-07-33809-s001.pdf]

Translin and Trax differentially regulate telomere-associated transcript homeostasis

Supplementary Material

Supplemental methods

Whole cell protein extraction and western blot analysis of TSN and TSNAX

SW480 cells were cultured in Invitrogen Dulbeco's Modified Eagle medium (DMEM + Glutamax) supplemented with 10% foetal bovine serum in 6 well plates and incubated at 37°C with 5% CO_2. Media was aspirated and a 200 µl volume of trypsin was added then incubated for 2 minutes at 37°C. 500 µl of medium was added and cells were transferred to an Eppendorf tube followed by centrifugation for 5 minutes at 3,000 *g*. The supernatant was aspirated and the cells washed with PBS. The pellet was resuspended in in an equal volume of lysis buffer [0.5% Triton-100, 200 mM NaCl, 50 mM Tris-HCL (pH 7.4), 1 mM AEBSF (Sigma, A8456) and a protease inhibitor table (Roche, 11836170001)] and loading buffer (Sigma, S3401). Samples were heated at 100°C for 5 minutes and loaded onto a pre-cast gel (Invitrogen, EC60352). The gel was run at 110 volts for approximately 45 minutes. An appropriate sized Hybond PVDF membrane was soaked in methanol and a blot was established in a transfer cassette in the following order: a Scotch Brite pad, two sheets of filter paper soaked in transfer buffer (0.2 M Tris, 1.92 M glysine), the gel, the membrane, two more sheets of filter paper soaked in transfer buffer and a Scotch Brite pad. The cassettes were placed in the transfer tank (with transfer buffer) and then run at 300 mA for approximately 1.5 hours. The membrane was removed and blocked using 1x PBS containing 10% skimmed powdered milk and 0.5% Tween for 1 hour on a shaker. The membrane was probed overnight with primary antibody in 1x PBS, 10% skimmed powdered milk and 0.5% Tween, washed for 5 minutes in 1x PBS and 0.5% Tween and then probed with secondary antibody. The membrane was then subjected to ECL detection (Thermo Scientific).
Primary antibodies used were: mouse anti-TNSAX (human) (Abcam, AB58642) at a dilution of 1:200; rabbit anti-TSN (human) (Abcam, AB71775) at a dilution of 1:500; mouse anti-Tubulin (human) (Sigma, T6074) at a dilution of 1:5000. Secondary antibodies used were: donkey anti-rabbit (Jackson Immuno Research, 711-005-152) at a dilution of 1:25,000; donkey anti-mouse (Jackson Immuno Research, 715-035-150) at a dilution of 1:25,000.

S. pombe *tiling array data analysis*

All tiling arrays were processed in R v3.0.1 [1] using Bioconductor packages. The quality of the arrays was assessed with the R packages preprocessCore [2], oligo [3], affy [4] and Starr [5]. The R package waveTiling was also used for background correction and quantile normalization. A batch effect was identified with PCA and removed using the ComBat function of the R package sva [6]. Current annotations (fungi_mart_26) were downloaded from Biomart using the R package GenomicFeatures [7]. Differentially expressed regions have been determined by means of the wavetiling R package and intersected with genome annotations. Genes with more than 15% overlap with significant regions as recommended in the waveTiling documentation, a log2FC > 1 and p value <

0.05 were considered significantly differentially expressed. Expression data was deposited in the National Center for Biotechnology Information Gene Expression Omnibus (http://www.ncbi.nlm.nih.gov/geo/) accession reference GSE71983.

1. R Core Team R: A language and environment for statistical computing. R Foundation for Statistical Computing, Vienna, Austria.URL http://www.R-project.org/
2. Bolstad BM preprocessCore*: A collection of pre-processing functions.* R package version 1.30.0.
3. Carvalho BS, Irizarry RA. A Framework for Oligonucleotide Microarray Preprocessing. Bioinformatics. 2010; 26: 2363-2367.
4. Gautier L, Cope L, Bolstad BM, Irizarry RA. affy—analysis of Affymetrix GeneChip data at the probe level. Bioinformatics. 2004; 20: 307–315.
5. Zacher B, Soeding J, Kuan PF, Siebert M, Tresch A. Starr: Simple tiling array analysis of Affymetrix ChIP-chip data. R package version 1.24.0 2009.
6. Leek JT. sva: Surrogate Variable Analysis. R package version 3.14.0.
7. Lawrence M, Huber W, Pagés H, Aboyoun R, Carlson M, Gentleman R, et al. Software for Computing and Annotating Genomic Ranges. PLoS Comput Biol. 2013; 9: e1003118.

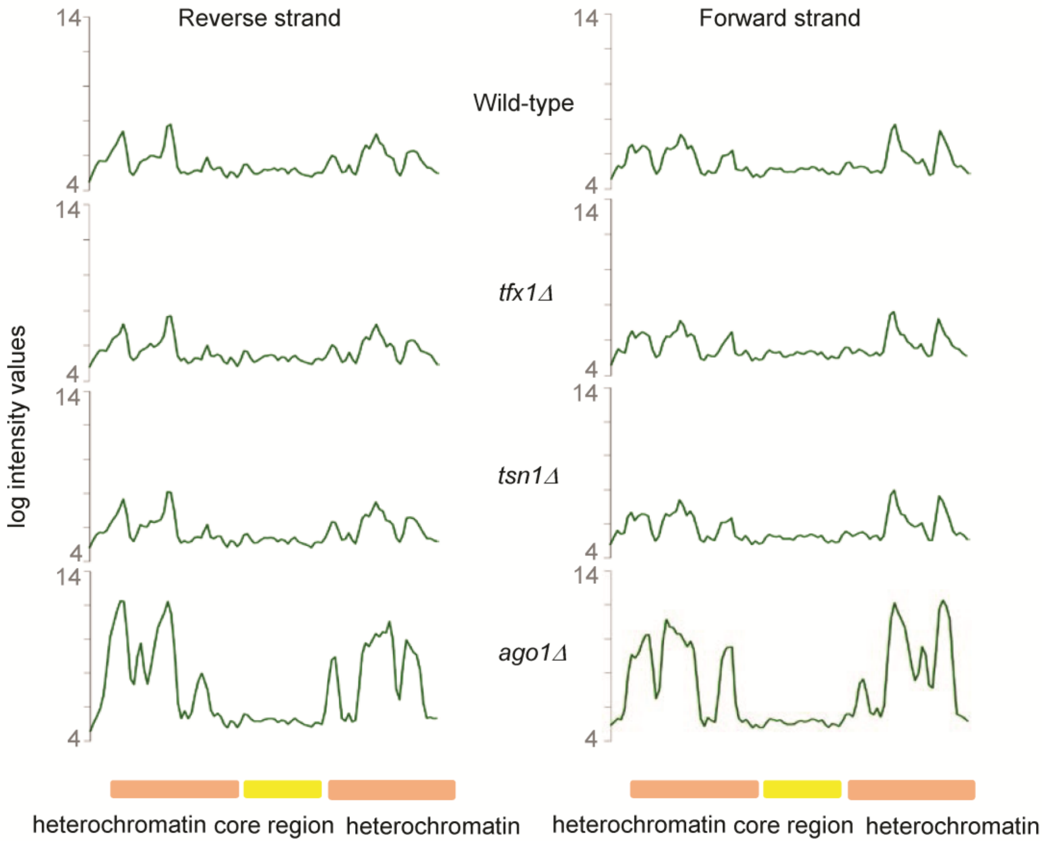

Fig. S1. Centromeric transcript silencing for the *cen2* forward (right) and reverse (left) strands remains unaltered in either *tfx1*Δ or *tsn1*Δ mutants (middle two profiles) compared to the wild-type (top profile). The profile for an *ago1*Δ mutant is given as a control for a desilencing mutant (bottom profile). The approximate spread of heterochromatic and centromere core regions are given (bottom line). The *S. pombe* nucleotide coordinates given for *cen2* are Chromosome 2: 1,600,000 – 1,645,000.

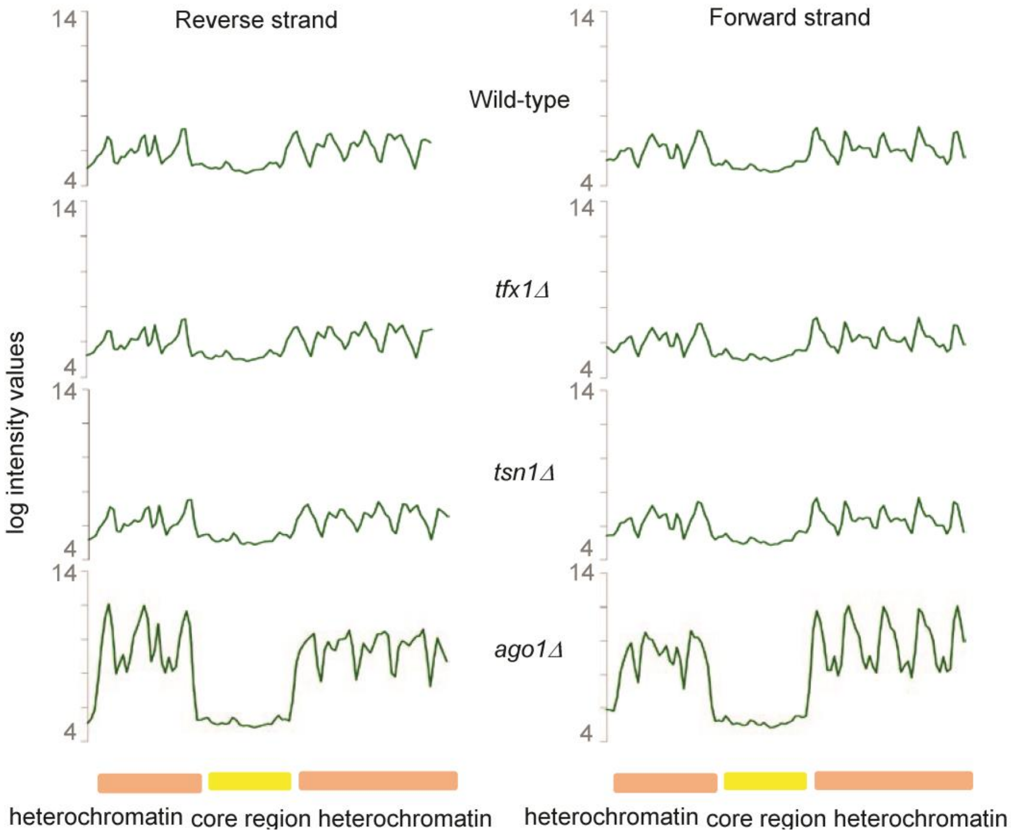

Fig. S2. Centromeric transcript silencing for the *cen3* forward (right) and reverse (left) strands remains unaltered in either *tfx1*Δ or *tsn1*Δ mutants (middle two profiles) compared to the wild-type (top). The profile for an *ago1*Δ mutant is given as a control for a desilencing mutant (bottom). The approximate spread of heterochromatic and centromere core regions are given (bottom). The *S. pombe* nucleotide coordinates given for *cen3* are Chromosome 3: 1,070,000 – 1,137,000.

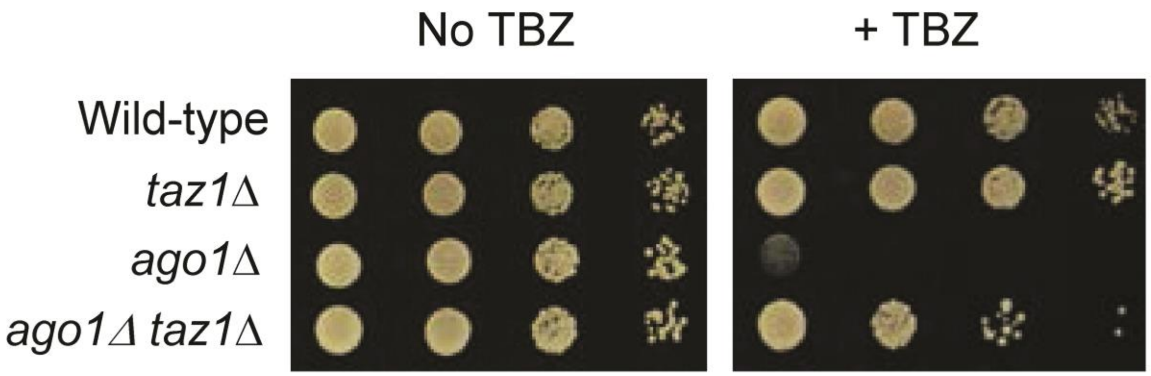

Fig. S3. Mutation of *taz1*$^{+}$ partially suppresses the TBZ sensitivity of an *ago1*Δ mutant.

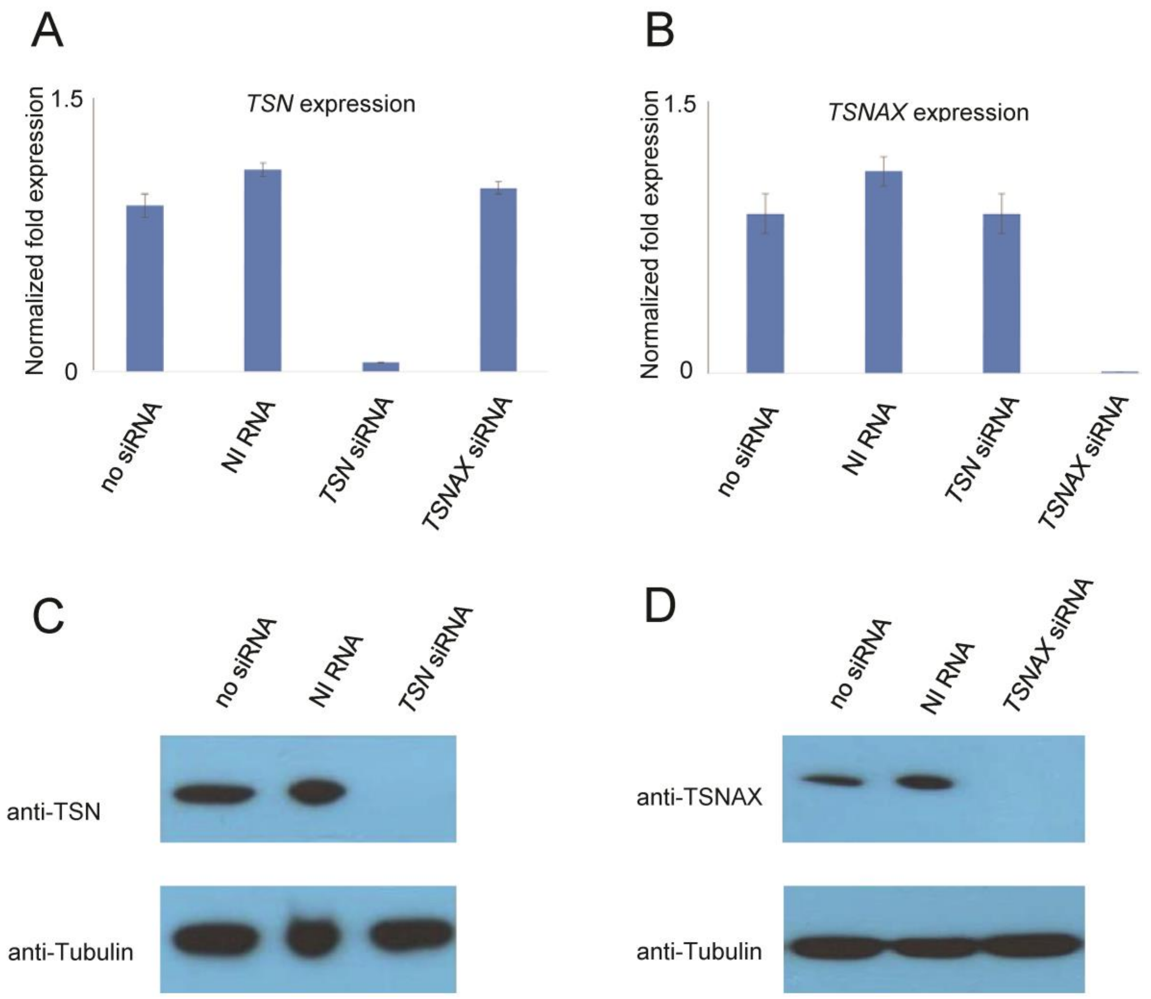

Fig. S4. Verification of the depletion of TSN and TSNAX in human SW480 cells. **A.** RT-qPCR analysis of *TSN* mRNA levels in *TSN* and *TSNAX* siRNA treated SW480 cells. **B.** RT-qPCR analysis of *TSNAX* mRNA levels in *TSN* and *TSNAX* siRNA treated SW480 cells. **C.** Western blot showing reduction of TSN levels following *TSN* siRNA treatment. **D.** Western blot showing reduction of TSNAX levels following *TSNAX* siRNA treatment.

Table S1. *Schizosaccharomyces pombe* strains employed in this study

Strain	Genotype	Source
BP90	*h⁻ ade6-M26 ura4-D18 leu1-32*	McFarlane Collection
BP118	*h⁻ ade6-M216 ura4-D18 leu1-32 taz1::ura4⁺*	McFarlane Collection
BP1080	*h⁻ ade6-M26 ura4-D18 leu1-32 tsn1::kanMX6*	This study
BP1089	*h⁻ ade6-M26 ura4 -D18 leu1-32 tfx1::kanMX6*	This study
BP2757	*h⁻ ade6-M26 ura4-D18 leu1-32 ago1::ura4⁺*	This study
BP2759	*h⁻ ade6-M26 ura4-D18 leu1-32 tsn1:: kanMX6 ago1::ura4⁺*	This study
BP2761	*h⁻ ade6-M26 ura4-D18 leu1-32 tfx1:: kanMX6 ago1::ura4⁺*	This study
BP3248	*h⁻ ade6-M26 ura4 -D18 leu1-32 tfx1::kanMX6 tsn1:: natMX6*	This study
BP3285	*h⁻ ade6-M26 ura4-D18 leu1-32 ago1::ura4⁺ taz1::natMX6*	This study
BP3301	*h⁻ ade6-M210 ura4-D18 leu1-32 his3-D1 Otrt::his3*	J. P. Cooper

Table S2. Primers used in this study

Primer designation	Primer sequence (5'-3')
Pombe oC	GTAACCCCTGTAACCGTAACCC
Pombe o3	GTGTGGAATTGAGTATGGTGAA
Pombe o2	GTGTAATACAGTAGTGCAGTG
Pombe o4	CGGCTGACGGGTGGGGCCCAATA
Pombe act1F	ATGGAAGAAGAAATCGCAG
Pombe act1R	CAAAACAGCTTGAATAGC
Human 10qF	GAATCCTGCGCACCGAGAT
Human 10qR	CTGCACTTGAACCCTGCAATAC
Human X/YqF	GGAAAGCAAAAGCCCCTCTGAATG
Human X/YqR	ACCCTCACCCTCACCCTAAGC
Human ACTF	TCCCTGGAGAAGAGCTACGA
Human ACTR	AGCACTGTGTTGGCGTACAG
Human TSNAX F	CCATCGAGCCATTACTACAG
Human TSNAX R	AGGTAATCGACAGGTGTGAC
Human TSN R	CTGTGAGCGAGATCTTCGTG
Human TSN F	TGCTGCCAAGAAGACCAAGC
